# HTCC: Broad Range Inhibitor of Coronavirus Entry

**DOI:** 10.1371/journal.pone.0156552

**Published:** 2016-06-01

**Authors:** Aleksandra Milewska, Kamil Kaminski, Justyna Ciejka, Katarzyna Kosowicz, Slawomir Zeglen, Jacek Wojarski, Maria Nowakowska, Krzysztof Szczubiałka, Krzysztof Pyrc

**Affiliations:** 1 Microbiology Department, Faculty of Biochemistry, Biophysics and Biotechnology, Jagiellonian University, Gronostajowa 7, 30-387 Krakow, Poland; 2 Malopolska Centre of Biotechnology, Jagiellonian University, Gronostajowa 7a, 30–387 Krakow, Poland; 3 Faculty of Chemistry, Jagiellonian University, Ingardena 3, 30-060 Krakow, Poland; 4 Department of Cardiac Surgery and Transplantology, Silesian Center for Heart Diseases, Marii Curie-Skłodowskiej 9, 41-800 Zabrze, Poland; German Primate Center, GERMANY

## Abstract

To date, six human coronaviruses have been known, all of which are associated with respiratory infections in humans. With the exception of the highly pathogenic SARS and MERS coronaviruses, human coronaviruses (HCoV-NL63, HCoV-OC43, HCoV-229E, and HCoV-HKU1) circulate worldwide and typically cause the common cold. In most cases, infection with these viruses does not lead to severe disease, although acute infections in infants, the elderly, and immunocompromised patients may progress to severe disease requiring hospitalization. Importantly, no drugs against human coronaviruses exist, and only supportive therapy is available. Previously, we proposed the cationically modified chitosan, N-(2-hydroxypropyl)-3-trimethylammonium chitosan chloride (HTCC), and its hydrophobically-modified derivative (HM-HTCC) as potent inhibitors of the coronavirus HCoV-NL63. Here, we show that HTCC inhibits interaction of a virus with its receptor and thus blocks the entry. Further, we demonstrate that HTCC polymers with different degrees of substitution act as effective inhibitors of all low-pathogenic human coronaviruses.

## Introduction

Coronaviruses (CoVs) are the largest group within the *Nidovirales* order, which is comprised of mammalian and avian pathogens. Based on their phylogenetic clustering, CoVs are sorted into four genera: Alpha-, Beta-, Gamma-, and Deltacoronaviruses, of which the Alpha- and Betacoronavirus genera encompass six human pathogens identified to date. CoVs are enveloped, positive-stranded RNA viruses with large genomes of approximately 27–32 kb, and their genomic RNA is capped and polyadenylated. The 5’ two-thirds of the CoV genome encode a polyprotein that is post-translationally processed to yield all proteins required for RNA replication (non-structural proteins), whereas the 3’ one-third of the genome encodes a canonical set of four structural proteins: spike (S), envelope, membrane, and nucleocapsid. The S glycoprotein of CoV belongs to class I viral fusion proteins and facilitates virus-receptor interactions. The envelope and membrane proteins form the virion shell, while the nucleocapsid protein interacts with viral RNA to form the virus core. Some species belonging to the Betacoronavirus genus encode an additional structural protein, hemagglutinin esterase (HE), which is thought to act as a receptor-degrading enzyme or mediates interaction with co-receptors, depending on the species [1].

All human CoVs (HCoVs) are associated with respiratory infections, two of which are known to cause severe, life-threatening diseases. The first, severe acute respiratory syndrome coronavirus (SARS-CoV), infected approximately 8,000 individuals in 2002–2003, with a mortality rate of nearly 10%. However, the virus disappeared before summer 2003 and never returned in its highly virulent form [2, 3]. The second, most recently isolated CoV, Middle East respiratory syndrome coronavirus (MERS-CoV) causes pneumonia and renal failure, with a mortality rate exceeding 30% [4, 5]. The remaining four low-pathogenic (LP) HCoVs, HCoV-229E, HCoV-NL63 (Alphacoronaviruses), HCoV-OC43, and HCoV-HKU1 (Betacoronaviruses), have been shown to circulate worldwide. Although infection with these viruses results primarily in the common cold in otherwise healthy adults, they may cause severe disease in young, elderly, and immunocompromised individuals [6–9].

To date, there are no effective drugs available to treat infection with HCoVs, including SARS-CoV and MERS-CoV. We previously proposed that the cationically modified chitosan, N-(2-hydroxypropyl)-3-trimethylammonium chitosan chloride (HTCC), and its hydrophobically-modified derivative (HM-HTCC) are potent inhibitors of HCoV-NL63 [10]. These polymeric compounds were able to impede replication of the virus *in vitro* in the permissive cell line LLC-MK2, as well as in an *ex vivo* model of fully differentiated human airway epithelium (HAE) primary cultures. Furthermore, these polymers were shown to inhibit murine hepatitis virus (MHV) infection in culture, suggesting that these chitosan-based compounds may represent a novel type of broad-spectrum inhibitors [10].

Here, we dissected the mechanism of the HTCC antiviral activity using functional assays, molecular methods, flow cytometry and confocal microscopy. A thorough analysis of the HTCC activity proved that the polymer interacts with the S protein of HCoV-NL63 and blocks its interaction with the cellular receptor, angiotensin-converting enzyme type 2 (ACE2) protein. Furthermore, we show that HTCC with different degrees of substitution (DSs) is to be a potent inhibitor of all four LP-HCoVs. DSs are expressed as the fraction of NH_2_ groups substituted within the chitosan chain. The DS of the studied HTCC polymers varied between 57% and 77%; thus the polymers were named HTCC-57, HTCC-62, HTCC-63, HTCC-65 and HTCC-77. The previously described HTCC polymer of a DS of 63% (here marked as HTCC-63) demonstrated an inhibitory effect not only on HCoV-NL63, but also on HCoV-OC43 and HCoV-HKU1. Interestingly, two different HTCCs, HTCC-62 and HTCC-77, proved to be effective inhibitors of HCoV-229E infection. Further, HTCC-65 effectively inhibited replication of HCoV-NL63 and HCoV-OC43, while HTCC-62 showed strong antiviral effect on HCoV-HKU1 infection. Taken together, the current analysis demonstrates that the combination of these cationically modified chitosan-based polymeric compounds into a single formulation may act as an effective broad-range anticoronaviral agent for the treatment of coronaviral infections.

## Materials and Methods

### Cell culture

LLC-Mk2 cells (ATCC: CCL-7; *Macaca mulatta* kidney epithelial) were maintained in minimal essential medium (MEM; two parts Hanks’ MEM and one part Earle’s MEM; Life Technologies, Poland) supplemented with 3% heat-inactivated fetal bovine serum (Life Technologies, Poland), penicillin (100 U/ml), streptomycin (100 μg/ml), and ciprofloxacin (5 μg/ml). Human HCT-8 cells (ATCC: CCL-244) were maintained in RPMI-1640 (Life Technologies, Poland) supplemented with 3% heat-inactivated fetal bovine serum (Life Technologies, Poland), penicillin (100 U/ml), streptomycin (100 μg/ml) and ciprofloxacin (5 μg/ml). Human MRC-5 (ATCC: CCL-171) cells were maintained in Dulbecco’s MEM (Life Technologies, Poland) supplemented with 10% heat-inactivated fetal bovine serum (Life Technologies, Poland), penicillin (100 U/ml), streptomycin (100 μg/ml), and ciprofloxacin (5 μg/ml). Cells were cultured at 37°C under 5% CO_2_.

### Human airway epithelium cultures

Human tracheobronchial epithelial cells were obtained from airway specimens resected from patients undergoing surgery under Silesian Center for Heart Diseases—approved protocols. This study was approved by the Bioethical Committee of the Medical University of Silesia in Katowice, Poland (approval no: KNW/0022/KB1/17/10 dated on 16.02.2010). Participants provided their written informed consent to participate in the study, as approved by the Bioethical Committee. Primary cells were expanded on plastic to generate passage 1 cells and plated on permeable Transwell inserts (6.5-mm-diameter) supports. Human airway epithelium (HAE) cultures were generated by provision of an air—liquid interface for 6–8 weeks to form well-differentiated, polarized cultures that resemble *in vivo* pseudostratified mucociliary epithelium.

### Synthesis of N-(2-hydroxypropyl)-3-trimethylammonium chitosan chloride (HTCC)

The synthesis of HTCC was performed as follows. 2.5 g of chitosan (molecular weight 250 ± 100 kDa, degree of deacetylation (DD) 83%) was dispersed in a mixture of 100 ml distilled water and 10 ml acetic acid. The solution was stirred for 30 min and varying volumes (5–8 ml) of glycidyltrimethylammonium chloride (GTMAC) were added to obtain the polymer with different DS values varying between 50 and 80%. The resulting mixture was heated and incubated at 55°C for 18 h while stirring under reflux condenser. The suspension was subsequently centrifuged at 4000 rpm for 10 min to remove suspended unreacted chitosan. The product was separated from the supernatant via precipitation in acetone and subsequent centrifugation at 4,000 rpm for 20 min. The solution was decanted and the resulting polymeric pellet was air dried and dissolved in distilled water. The purification process was repeated twice and the purified HTCC was dried in vacuum oven for 24 h. The DSs of the polymers obtained are given in Table 1.

**Table 1:** The HTCC polymers used in the studies.

Polymer	Molecular mass	DS (%)
HTCC-57	250 ± 100kDa	57
HTCC-62	250 ± 100kDa	62
HTCC-63	250 ± 100kDa	63
HTCC-65	250 ± 100kDa	65
HTCC-77	250 ± 100kDa	77

### Virus preparation and titration

The HCoV-NL63 stock (isolate Amsterdam 1) was generated by infecting monolayers of LLC-Mk2 cells. The HCoV-OC43 (ATCC: VR-1558) and HCoV-229E (NCPV: 0310051v) stocks were generated by infecting monolayers of HCT-8 and MRC-5 cells, respectively. Cells were then lysed by two freeze-thaw cycles at day 6 post-infection (p.i.). The virus-containing liquid was aliquoted and stored at −80°C. Control LLC-Mk2, HCT-8 and MRC-5 cell lysates from mock-infected cells were prepared in the same manner. Each virus yield was assessed by titration on fully confluent LLC-Mk2, HCT-8 or MRC-5 cells in 96-well plates, according to the method of Reed and Muench [11]. Plates were incubated at 32°C for 6 days and the cytopathic effect (CPE) was scored by observation under an inverted microscope. HCoV-OC43 (isolate 0500) replication was analyzed by real-time RT-PCR. HCoV-HKU1 (strain Caen 1) was propagated on fully differentiated HAE cultures, as previously described [12]. The virus replication was analyzed by real-time RT-PCR.

### Gradient purification of HCoV-NL63

The virus stock was concentrated 25-fold using centrifugal protein concentrators (Amicon Ultra, 10 kDa cut-off; Merck, Poland) and subsequently overlaid on 15% iodixanol solution in 1 × PBS (OptiPrep medium; Sigma-Aldrich, Poland). Following virus concentration using iodixanol cushion (centrifugation at 175 000 × g for 3 h at 4°C), it was overlaid on 10-20% iodixanol gradient (in 1 × PBS) and centrifuged at 175 000 × g for 18 h at 4°C. Fractions (1 ml) collected from the gradient were analyzed by Western blotting, followed by immunodetection of the HCoV-NL63 nucleocapsid protein. The virus-containing fractions were aliquoted and stored at −80°C. The control cell lysate (mock) was concentrated and prepared in the same manner as the virus stock.

### Virus infection

In subsequent *in vitro* experiments, fully confluent HCT-8, MRC-5 or LLC-Mk2 cells in 96-well plates (TPP) were exposed to HCoV-OC43, HCoV-229E or HCoV-NL63, respectively, at a TCID_50_ of 400 in the presence of tested polymer or control medium (MEM for HCoV-NL63 and DMEM for HCoV-OC43 and HCoV-229E). Following a 2 h incubation at 32°C, unbound virions were removed by washing with 100 μl of 1 × PBS and fresh medium containing dissolved respective polymer was added to each well. Samples of cell culture supernatant were collected at day 5 p.i. and analyzed by real-time RT-PCR.

For the *ex vivo* study, fully differentiated human airway epithelium (HAE) cultures were exposed to the tested polymer or control PBS for 30 min at 37°C, following inoculation with HCoV-NL63, HCoV-229E, HCoV-OC43 or HCoV-HKU1 in the presence of the polymer or control PBS. Following 2 h incubation at 32°C, unbound virions were removed by washing with 100 μl of 1 × PBS and HAE cultures were maintained at an air—liquid interphase for the rest of the experiment. To analyze virus replication kinetics, each day p.i., 100 μl of 1 × PBS was applied at the apical surface of HAE and collected following the 10 min incubation at 32°C. All samples were stored at −80°C and analyzed by real-time RT-PCR.

### Cell viability assay

LLC-Mk2, MRC-5 and HCT-8 cells were cultured on 96-well plates, as described above. Cell viability assay was performed by using the XTT Cell Viability Assay (Biological Industries, Israel), according to the manufacturer’s instructions. On the day of the assay the medium was removed and 100 μl of the culture medium with 30 μl of the activated XTT solution was added to each well. Following 2 h incubation at 37°C, the medium was transferred onto a new 96-well plate and signal was measured at λ = 490 nm using the colorimeter (Spectra MAX 250, Molecular Devices). The obtained results were further normalized to the control sample, where cell viability was set to 100%.

For the assessment of the cell viability in human airway epithelium (HAE) cultures, the apical surface was washed with 100 μl of 1 × PBS and 100 μl of 1 × PBS with 50 μl of the activated XTT solution was applied. Plates were then incubated at 37°C for 2 h, apical liquid was transferred to the new 96-well plate and the signal was measured, as described above.

### Isolation of nucleic acids and reverse transcription

Viral nucleic acids were isolated from cell culture supernatants using the Total RNA Mini-Preps Super Kit (Bio Basic, Canada), according to the manufacturer’s instructions. Reverse transcription was carried out with a High Capacity cDNA Reverse Transcription Kit (Life Technologies, Poland), according to the manufacturer’s instructions.

### Quantitative RT-PCR

Virus yields were determined using real-time PCR (7500 Fast Real-Time PCR machine; Life Technologies, Poland). Viral cDNA (2.5 μl per sample) was amplified in a 10 μl reaction mixture containing 1 × TaqMan Universal PCR Master Mix (Life Technologies, Poland), specific probes labeled with 6-carboxyfluorescein (FAM) and 6-carboxytetramethylrhodamine (TAMRA) (100 nM), and primers (450 nM each). Rox was used as the reference dye. All primers and probes are listed in Table 2. The reaction conditions were as follows: 2 min at 50°C and 10 min at 92°C, followed by 40 cycles of 15 sec at 92°C and 1 min at 60°C. In order to assess the copy number for each HCoVs, RNA standards were prepared. Briefly, N genes of HCoV-OC43, HCoV-229E, HCoV-HKU1 and HCoV-NL63 were amplified and cloned into pTZ57R/T (Thermo Scientific, Poland) plasmids using InsTAclone PCR cloning kit (Thermo Scientific, Poland). Subsequently, DNA vectors were amplified and linearized with *Eco*RI restriction enzyme. Linear nucleic acids were further purified with the GeneJET^™^ PCR Purification Kit (Thermo Scientific, Poland) and used as a template for *in vitro* transcription using T7 RNA polymerase (Thermo Scientific, Poland). Reaction was conducted according to the manufacturer’s instructions. Resulting RNA was DNase treated (DNase Turbo; Life Technologies, Poland) and purified with the Total RNA Mini-Preps Super Kit (Bio Basic, Canada), according to the manufacturer’s instructions and its concentration was assessed using a spectrophotometer. The number of RNA copies/ml was assessed using Avogadro’s constant and molecular mass of RNA molecules. Samples were serially diluted and used as an input for reverse transcription and real-time PCR reaction.

**Table 2:** Primers and probes used for a quantitative RT-PCR.

Target species	Primer	Primer sequence (5’− 3’)
HCoV-OC43	Sense (OC43F_rt)	AGCAACCAGGCTGATGTCAATACC
	Antisense (OC43R_rt)	AGCAGACCTTCCTGAGCCTTCAAT
	Probe (OC43P_rt)	TGACATTGTCGATCGGGACCCAAGTA
HCoV-229E	Sense (229E_NF)	GTTGTGGCCAATGGTGTTAAAG
	Antisense (229E_NR)	AGTGTTGCCTGACTCTTTGG
	Probe (229E_NP)	ACAATTTGCTGAGCTTGTGCCGTC
HCoV-HKU1	Sense (HKUqPCR5)	CTGGTACGATTTTGCCTCAA
	Antisense (HKUqPCR3)	ATTATTGGGTCCACGTGATTG
	Probe (HKUqPCRP)	TTGAAGGCTCAGGAAGGTCTGCTTCTAA
HCoV-NL63	Sense (63NF2)	CTGTGGAAAACCTTTGGCATC
	Antisense (NR1)	CTGTGGAAAACCTTTGGCATC
	Probe (63NP)	ATGTTATTCAGTGCTTTGGTCCTCGTGAT

### Flow cytometry

LLC-Mk2 cells were seeded on 6-well plates (TPP) and cultured for 2 days at 37°C with 5% CO_2_. For visualization of adhered HCoV-NL63 particles, cells were washed with 1 × PBS and incubated with iodixanol—concentrated HCoV-NL63 or mock samples in the presence of HTCC-63 (200 μg/ml) or control PBS for 2 h at 4°C. Subsequently, cells were washed with sterile PBS, fixed with 3% PFA, permeabilized with 0,1% Triton X100 in 1 × PBS and incubated for 1 h with 3% BSA in 1 × PBS with 0.1% Tween 20. To assess the HCoV-NL63 adhesion, cells were scraped from plastic and incubated for 2 h at room temperature with mouse anti-HCoV-NL63-N antibody (1 μg/ml, Ingenansa, Spain), followed by 1 h incubation with Alexa Fluor 488-labeled goat anti-mouse antibody (2.5 μg/ml, Molecular Probes). Cells were then washed, re-suspended in 1 × PBS and analyzed with FACS Calibur (Becton Dickinson) using Cell Quest software.

### Confocal microscopy

LLC-Mk2 cells were seeded on coverslips in 6-well plates (TPP) and cultured for 2 days at 37°C. Subsequently, cells were washed with 1 × PBS and incubated with iodixanol-concentrated HCoV-NL63 or mock samples in the presence of the HTCC-63 (200 μg/ml) or control 1 × PBS for 2 h at 4°C. Cells were then washed with 1 × PBS, fixed with 3% PFA, permeabilized with 0.1% Triton X-100 in 1 × PBS and incubated overnight at 4°C with 1 × PBS supplemented with 10% BSA and 0.5% Tween 20. To visualize adhered HCoV-NL63 particles, cells were incubated for 2 h at room temperature with mouse anti-HCoV-NL63-N IgG (0.25 μg/ml; Ingenansa, Spain), followed by a 1 h incubation with Alexa Fluor 488-labeled goat anti-mouse IgG (2.5 μg/ml, Life Technologies, Poland). For ACE2 staining, cells were incubated for 2 h at room temperature with goat anti-ACE2 ectodomain IgG (4 μg/ml; R&D Systems, USA), followed by a 1 h incubation with Alexa Fluor 546-labeled donkey anti-goat IgG (2.5 μg/ml, Life Technologies, Poland). Nuclear DNA was stained with DAPI (0.1 μg/ml, Sigma-Aldrich, Poland). Immunostained cultures were mounted on glass slides in ProLong Gold antifade medium (Thermo Scientific, Poland). Fluorescent images were acquired under a Leica TCS SP5 II confocal microscope (Leica Microsystems GmbH, Mannheim, Germany). Images were acquired using Leica Application Suite Advanced Fluorescence LAS AF v. 2.2.1 (Leica Microsystems CMS GmbH), deconvolved with Huygens Essential package ver. 4.4 (Scientific Volume Imaging B.V.; The Netherlands) and processed using ImageJ 1.47v (National Institutes of Health, Bethesda, Maryland, USA).

### Statistical analysis

All the experiments were performed in triplicate and the results are presented as mean ± SD. To determine significance of the obtained results, a comparison between groups was conducted using the one-way ANOVA test. *P* values < 0.05 were considered significant.

## Results

### Mechanism of the HTCC antiviral activity

The first aim of the study was to investigate the mechanism of action of the previously described HTCC-63 polymer. For this, we analyzed the influence of HTCC-63 on different steps of HCoV-NL63 replication cycle. First, we determined whether the polymer interacts directly with HCoV-NL63 virions. To achieve this, concentrated virus stock was incubated with the polymer (200 μg/ml) or control 1 × PBS at room temperature for 2 h. Subsequently, samples were diluted 200 times (polymer concentration was below the active range) and titrated as described by Reed and Muench; titers were evaluated on day 5 p.i. [11]. No decrease in virus titer was observed for the HTCC-63 polymer, compared to control sample (data not shown).

**Fig 1:**
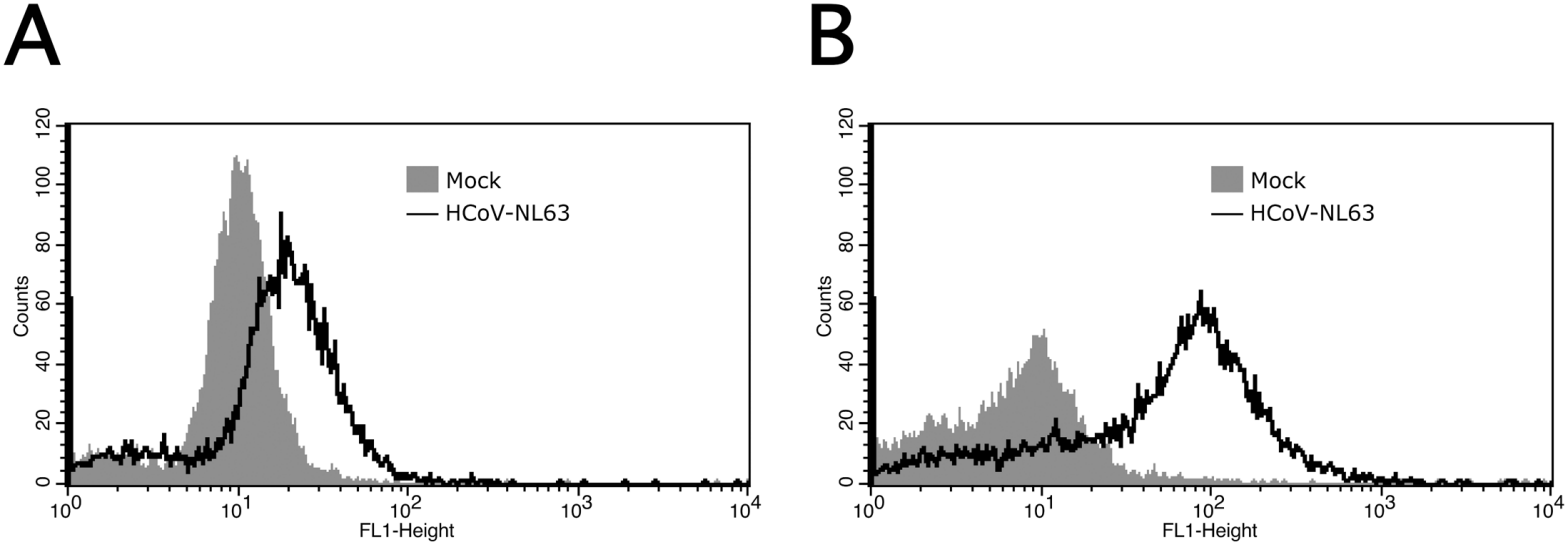
HTCC-63 does not affect HCoV-NL63 attachment to target cells. LLC-Mk2 cells were incubated with gradient-purified HCoV-NL63 or mock in the presence of control PBS **(A)** or HTCC-63 at 200 μg/ml **(B)** for 2 h at 4°C. Analysis of HCoV-NL63 adherence was conducted with flow cytometry using specific antibodies. The data shown are representative of at least three independent experiments, each performed in duplicate.

Further, the effect of HTCC-63 on virus adhesion to susceptible cells was evaluated. Briefly, LLC-Mk2 cells were pre-cooled and incubated with gradient-purified HCoV-NL63 at 4°C for 2 h in the presence of HTCC-63 (200 μg/ml) or control PBS. At this temperature virus can bind to the receptor, while virus internalization is suppressed due to intracellular trafficking inhibition. Next, HCoV-NL63 cell surface adhesion was evaluated using flow cytometry. The virus adherence was not inhibited in the presence of HTCC-63, suggesting that the polymer does not hamper virus binding to heparan sulfate proteoglycans (HSPs) (Fig 1) [13]. Surprisingly, the adherence of HCoV-NL63 particles seemed to be enhanced in the presence of HTCC. One may assume that HCoV-NL63 virions cluster in the presence of HTCC, as they bind to the polymer *via* the S protein forming aggregates. To support these data, another assay was performed, where LLC-Mk2 cells were pre-cooled and incubated with HCoV-NL63 (TCID_50_ of 400) at 4°C for 1 h in the presence of HTCC-63 (200 μg/ml) or control PBS. Next, culture media containing the unbound virus were removed and cells were rinsed 3 times with ice cold 1 × PBS. Virus infection was evaluated at day 5 p.i., based on the occurrence of the cytopathic effect (CPE) and quantitative RT-PCR. Obtained results demonstrated that HTCC-63 did not inhibit adhesion of HCoV-NL63 virions to the cell surface (data not shown).

**Fig 2:**
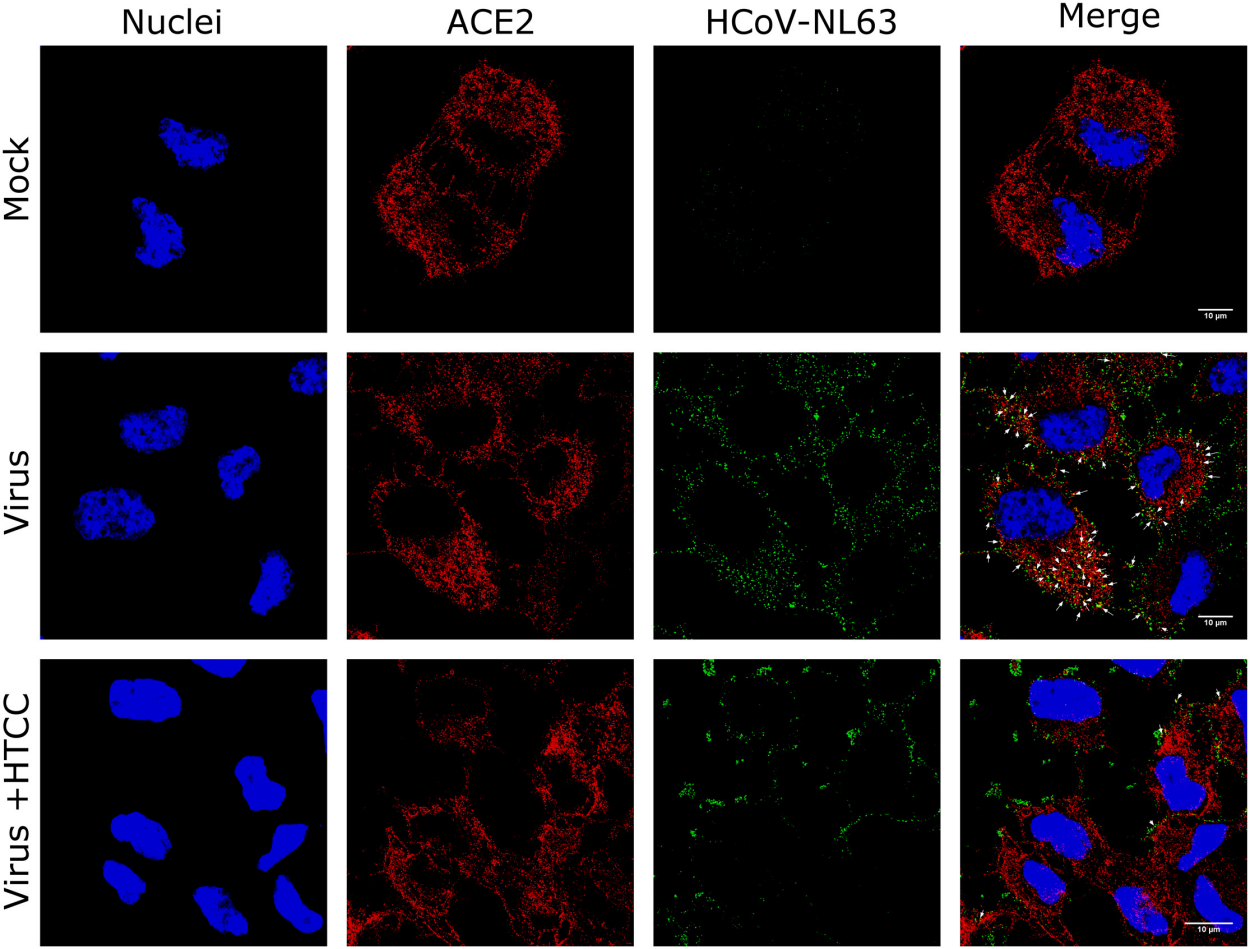
HTCC-63 blocks HCoV-NL63 binding to the ACE protein. LLC-Mk2 cells were incubated with gradient-purified HCoV-NL63 in the presence of control PBS (Virus) or HTCC-63 at 200 μg/ml (Virus+HTCC) or mock. HCoV-NL63 interaction with the ACE2 protein was analyzed with confocal microscopy. Each image represents maximum projection of axial planes. Virus interaction with the ACE2 was marked with white arrows.

Whereas HSPs establish primary binding sites for HCoV-NL63, the virus requires presence of the functional receptor (ACE2) [14]. In the next step we aimed to test whether HTCC-63 blocks interaction between HCoV-NL63 and ACE2. For this, we analyzed virus co-localization with the receptor in the presence of the HTCC-63 or control 1 × PBS. As naturally susceptible LLC-Mk2 cells express only low levels of the ACE2 protein, we used previously described A549 cells with directed ACE2 expression (A549_ACE2^+^) [13]. Briefly, A549_ACE2^+^ cells were incubated at 4°C with gradient-purified HCoV-NL63 in the presence of HTCC-63 (200 μg/ml) or control PBS. Subsequently, cells were fixed and immunostained for the ACE2 and HCoV-NL63 nucleoprotein using specific antibodies. Virus co-localization with its receptor was examined using confocal microscopy. Obtained results demonstrated that in the control samples virions co-localize with the ACE2 protein, while in the presence of the polymer this interaction is abolished (Fig 2). This result is consistent with our previous observations that HTCC-63 binds to the S protein of HCoV-NL63 [10].

**Fig 3:**
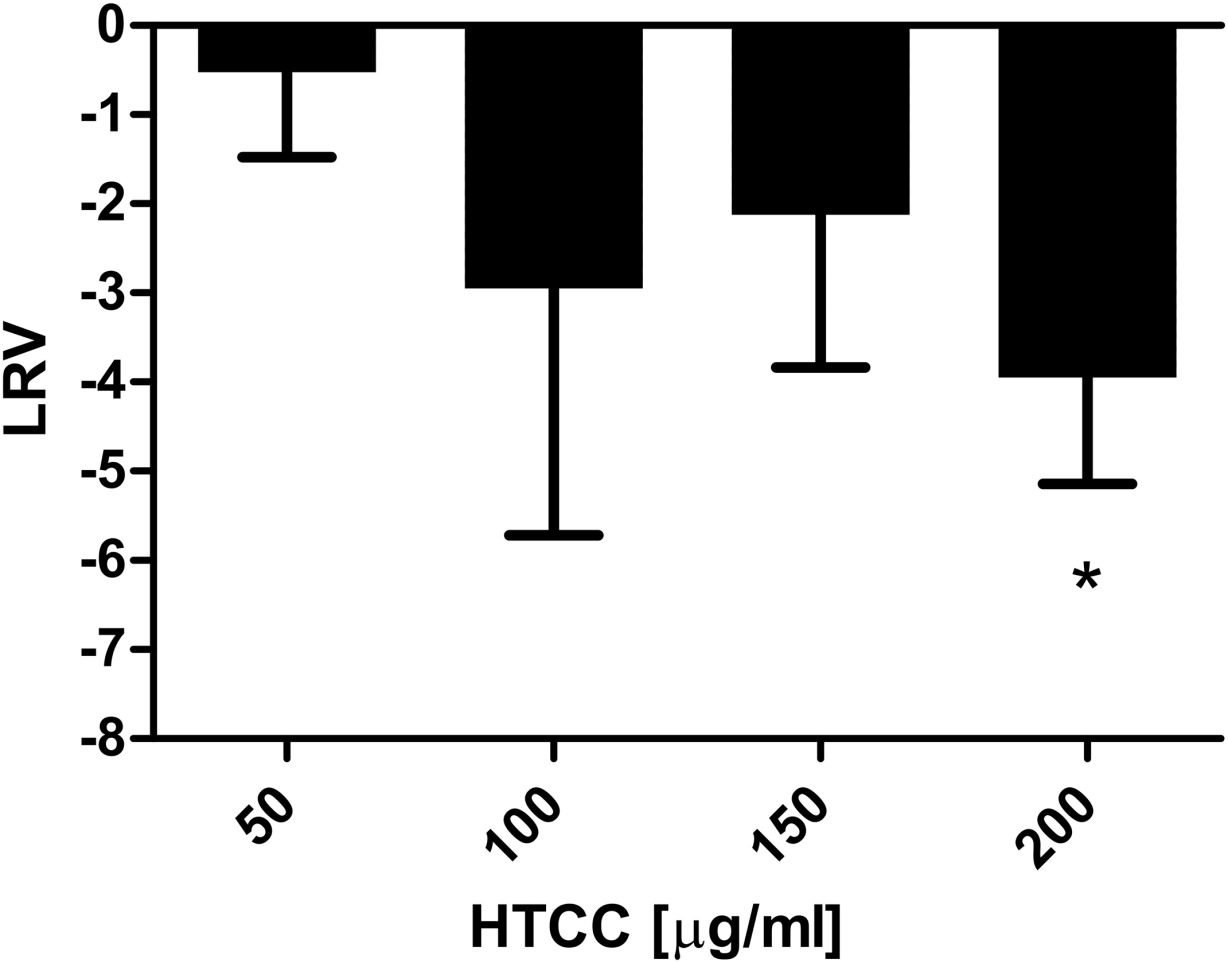
HCoV-NL63 internalization into susceptible cells is hampered by HTCC. Pre-cooled LLC-Mk2 cells were incubated with cold HCoV-NL63 at 4°C, followed by incubation with HTCC (50–200 μg/ml) or control PBS for 2 h at 32°C. Next, unbound virus was removed by washing with acidic buffer and cells were further incubated at 32°C for 5 days. Virus yield was determined with quantitative RT-PCR and is presented as Log Reduction Value (LRV). The data shown are representative of at least three independent experiments, each performed in triplicate. * *P* < 0.05.

To ensure that blocking HCoV-NL63 interaction with the ACE2 is the main mechanism of the HTCC antiviral activity, we analyzed the effect using also the functional assay. First, we analyzed whether the polymer affects HCoV-NL63 internalization to susceptible cells, for which virion-ACE2 interaction is indispensable. Briefly, pre-cooled LLC-Mk2 cells were inoculated with ice-cold HCoV-NL63 at TCID_50_ of 400 and incubated at 4°C for 2 h to allow virus binding, but not internalization. Next, virus particles were removed by washing with ice-cold 1 × PBS and cells were incubated with the HTCC-63 (50–200 μg/ml) or control medium at 32°C for 2 h to enable virus penetration and to evaluate whether the polymer interferes with this process. Subsequently, the media were removed and cells were washed 3 times with acidic buffer (0.1 M glycine, 0.1 M NaCl, pH 3.0) in order to inhibit the fusogenic activity of virions that were not internalized [15–17]. The efficiency of virus deactivation using the low pH was verified prior to these experiment (data not shown). Subsequently, cells were washed with 1 × PBS (pH 7.4), and overlaid with culture medium. Cells were incubated at 32°C for 5 days. The occurrence of the CPE at day 5 p.i. was evaluated and virus yield in cell culture supernatants was measured using a quantitative RT-PCR. First, no CPE was visible in cultures incubated with the polymer, while in control culture obvious signs of virus replication were visible. qRT-PCR analysis confirmed these qualitative results, as 4-log decrease in virus yield was observed in samples treated with HTCC-63 polymer, compared to control cells (Fig 3).

### Inhibition of HCoVs replication in HAE

**Fig 4:**
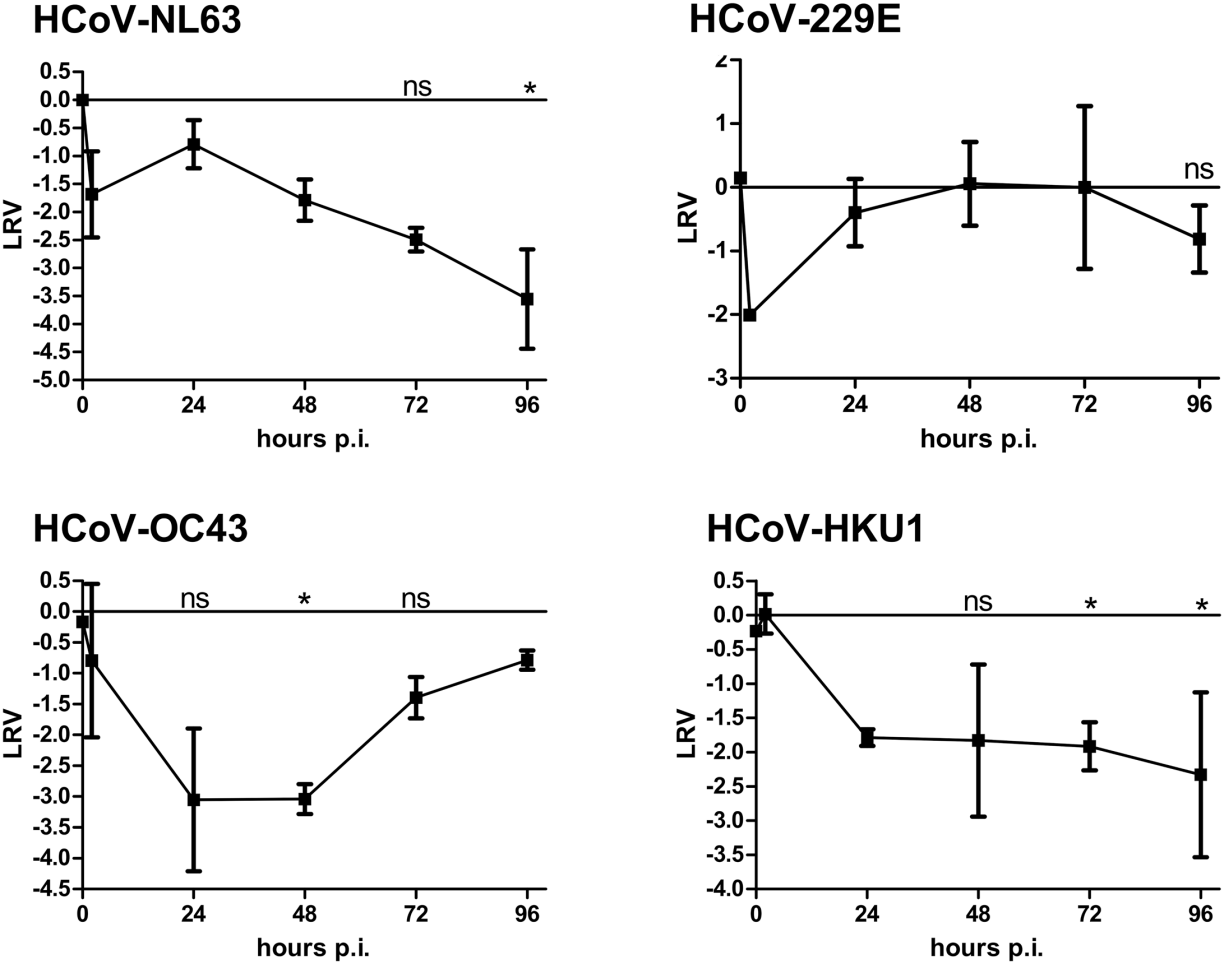
HTCC-63 inhibits HCoV-NL63, HCoV-OC43, and HCoV-HKU1 replication in human airway epithelium (HAE) cultures. **(A)** HAE cultures were exposed to HCoV-NL63, HCoV-OC43, HCoV-229E, or HCoV-HKU1 in the presence of HTCC-63 (200 μg/ml) or control PBS. To analyze virus replication kinetics, each day post infection, 100 μl of 1× PBS was applied to the apical surface of HAE cultures and collected after 10 min of incubation at 32°C. Replication of viruses was evaluated using quantitative RT-PCR. The data are presented as Log Removal Value (LRV) compared to the untreated sample. The assay was performed in triplicate, and average values with standard errors are presented. * *P* < 0.05; ns = not significant.

The next aim of the study was to evaluate the antiviral activity of the previously described HTCC-63 polymer against other LP-HCoVs. For this, we employed primary HAE cultures, which mimic the natural respiratory tract microenvironment. Briefly, fully differentiated HAE cultures were infected with HCoV-NL63, HCoV-229E, HCoV-OC43, or HCoV-HKU1 in the presence of HTCC-63 (200 μg/ml) or control PBS. Following inoculation, apical lavage samples were collected daily, and replication kinetics for each virus were investigated. Analysis revealed that the polymer hampered infection by HCoV-NL63, HCoV-OC43, and HCoV-HKU1, but did not affect HCoV-229E replication. For HCoV-OC43, the inhibitory effect was the most evident until 72 h p.i., while the effect on HCoV-NL63 and HCoV-HKU1 replication was permanent (Fig 4). Importantly, no significant cytotoxicity was observed by XTT assay, confirming that the observed inhibitory effect did not result from HTCC-63-induced cytotoxicity (S1 Fig).

### Synthesis of HTCC compounds with different DS

Five cationic derivatives of chitosan were obtained (Table 1) by reacting chitosan with GTMAC. The substitution of chitosan free amino groups with the GTMAC moiety, which contains an amino group, imparted a stable (i.e., independent of pH) positive charge on the chitosan macromolecule and improved its solubility at neutral pH. The DS of each compound was determined using a titration method described and validated in the literature. The method is based on the conductimetric titration of chloride ions present in the polymer using silver nitrate solution [18, 19].

### Inhibition of HCoVs replication by HTCC with different DS

The second aim of this study was to investigate how different DSs affect the effectiveness. To analyze the effect of different variants of HTCC on HCoV-NL63, HCoV-229E, HCoV-OC43, and HCoV-HKU1 infection, a series of experiments was conducted. Taking into consideration the cost-effectiveness and limited availability of primary cultures, three out of the four experiments were performed *in vitro*, using the following permissive cell lines: LLC-Mk2 (for HCoV-NL63), MRC-5 (for HCoV-229E), and HCT-8 (for HCoV-OC43). The antiviral effect of the HTCC polymers against HCoV-HKU1 infection was investigated using the *ex vivo* HAE model, as there is no permissive cell line available for this virus [12].

**Fig 5:**
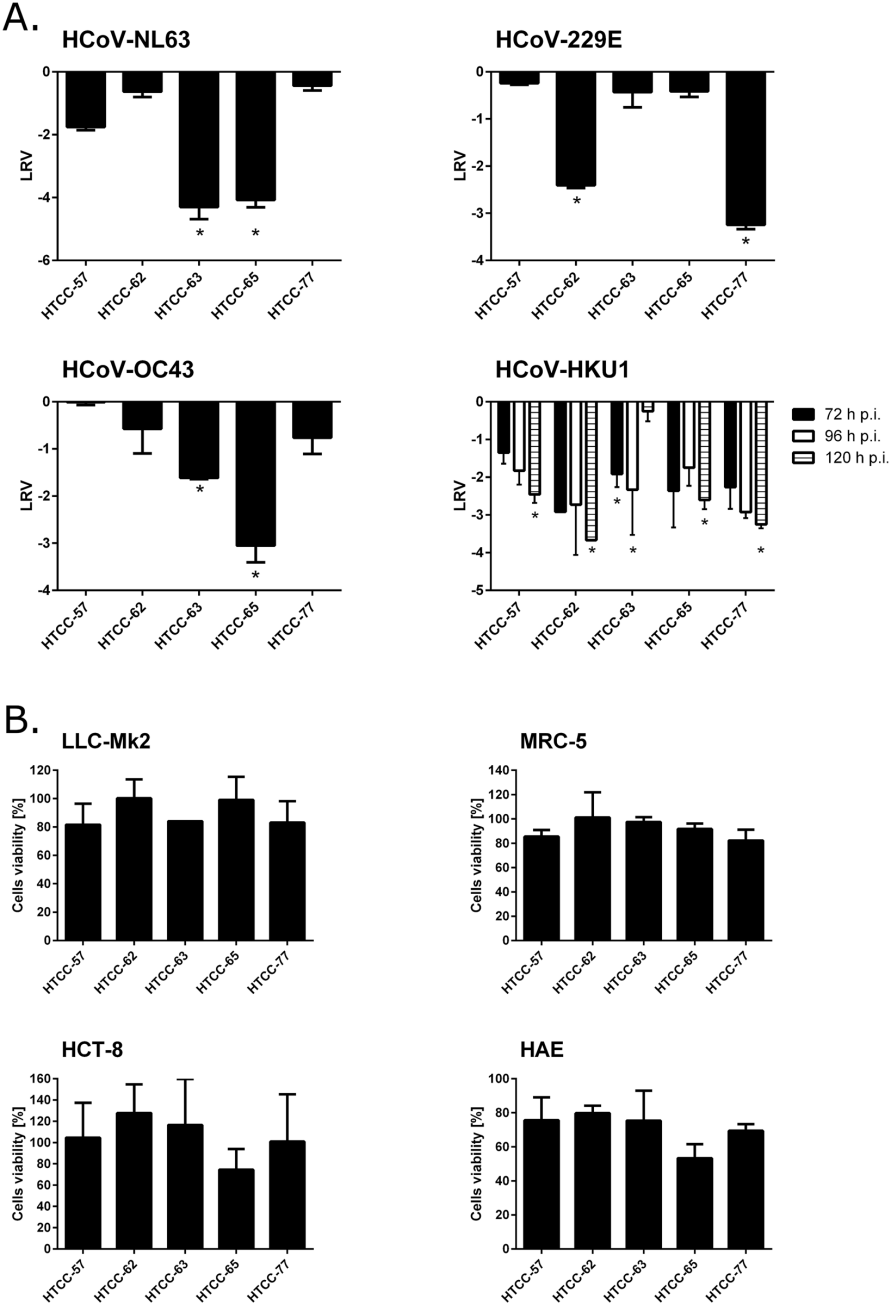
HTCCs with different DS effectively inhibit HCoV-NL63, HCoV-229E HCoV-OC43, and HCoV-HKU1 infection. LLC-Mk2 cells were infected with HCoV-NL63 in the presence of HTCCs with DS ranging from 57% to 77% at a concentration of 200 μg/ml or control medium. Likewise, MRC-5 cells were infected with HCoV-229E and HCT-8 cells were infected with HCoV-OC43 in the presence of HTCCs with DS ranging from 57% to 77% at 200 μg/ml or control medium. HAE cultures were infected with HCoV-HKU1 in the presence of HTCCs with DS ranging from 57% to 77% (200 μg/ml) or PBS (control). **(A)** Virus replication in cell culture supernatants (cell lines) or apical lavages (HAE) was evaluated using quantitative RT-PCR. Data are presented as Log Removal Value (LRV) compared to untreated samples. The assays were performed in triplicate, and average values with standard errors are presented. * *P* < 0.05. **(B)** Cytotoxicity of HTCCs with DS ranging from 57% to 77% in cell lines and HAE cultures. Cell viability was assessed via XTT assay. Data on the y-axis represent the percentage values obtained for the untreated reference samples. All assays were performed in triplicate in at least two independent experiments, and average values with standard errors are presented. The differences in cytotoxicity of HTCCs were not statistically significant.

For the *in vitro* experiments, cells seeded in monolayer on 96-well plates were infected with a respective CoV at a TCID_50_ of 400 in the presence of HTCCs with various DS (200 μg/ml each) or control media. After incubation for 5 days at 32°C, the reduction in CPE in susceptible cells was investigated, and virus yields were analyzed in the cell culture supernatants. The analysis revealed that infection of each studied pathogen was effectively inhibited by at least two compounds. HCoV-NL63 replication in LLC-Mk2 cells was reduced not only by the previously described HTCC-63, but also by HTCC-65. Similarly, HCoV-OC43 infection in HCT-8 cells was effectively inhibited by HTCC-63 and HTCC-65, with a more prominent inhibitory effect observed for HTCC-65 (an approximate 3 log reduction in virus yield compared to the control sample). On the other hand, HTCCs of two different DSs (HTCC-62 and HTCC-77) inhibited HCoV-229E infection in MRC-5 cells with comparable effectiveness (an approximate 3 log reduction in virus yield compared to the control sample). No inhibition of HCoV-229E infection was observed for HTCC-57, HTCC-63, or HTCC-65 (Fig 5A).

To study the effect of HTCCs on HCoV-HKU1 infection of HAE, cultures were infected with the virus at a concentration of 10^4^ RNA copies/ml in the presence of selected HTCC polymers or control PBS. Unfortunately, titration of this virus was not possible due to the lack of appropriate *in vitro* culture models. Analysis revealed that all of the studied HTCC polymers affected HCoV-HKU1 infection, with HTCC-62 and HTCC-77 being the most effective. At 72–120 h p.i., a 3–4 log decrease in the HCoV-HKU1 yield was observed in samples supplemented with HTCC-62 or HTCC-77 compared to the control (Fig 5A).

To ensure that the observed effect did not result from a cytotoxic effect of the polymers, XTT assays were performed for each experimental setting. The analysis clearly showed that the viability of the cell lines or HAE cultures was not significantly affected by the tested polymers at tested concentrations (Fig 5B).

### The combination of HTCCs with different DS exhibits broad anticoronaviral activity

**Fig 6:**
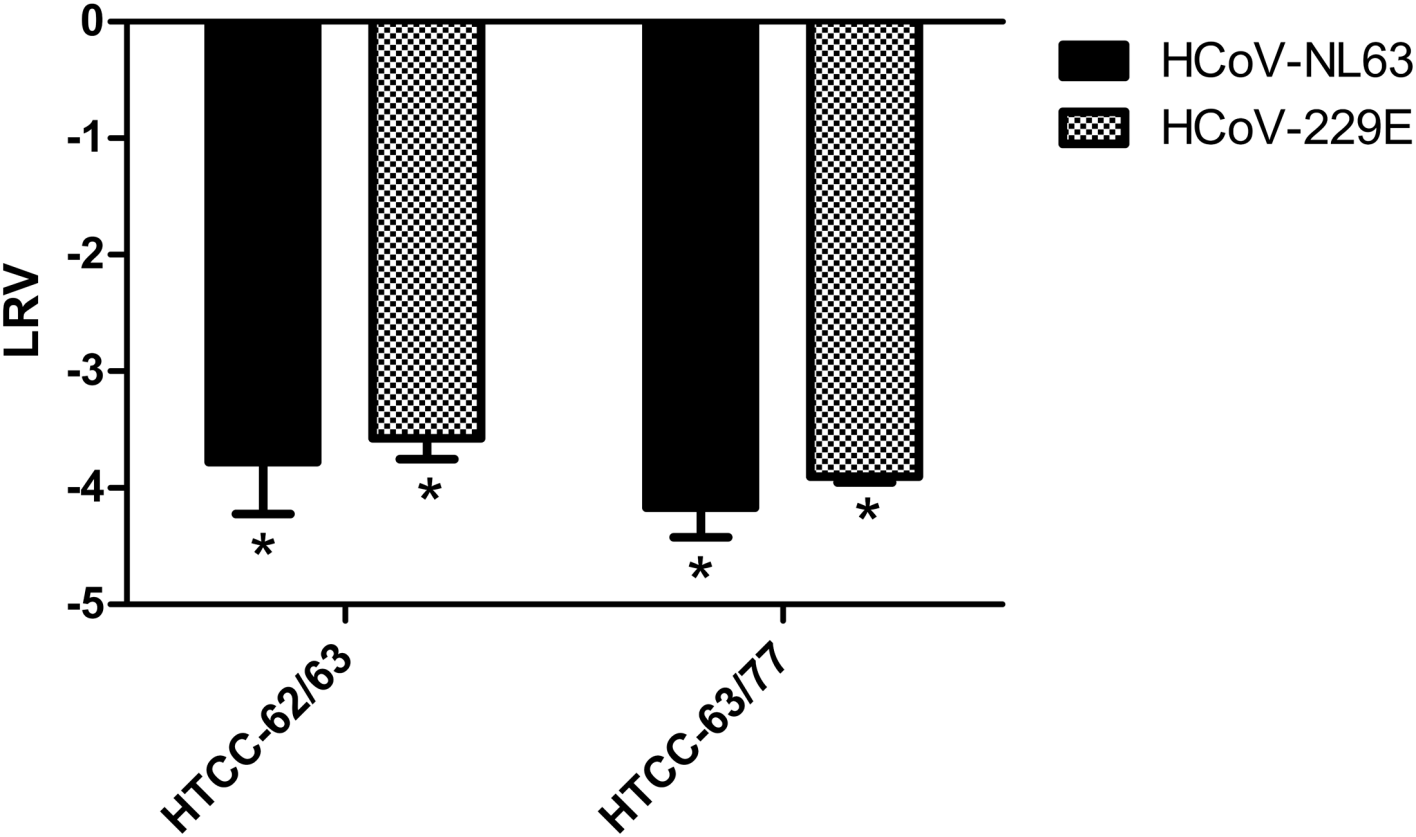
HTCC-62/63 and HTCC-63/77 mixtures hamper replication of HCoV-229E and HCoV-NL63. MRC-5 and LLC-Mk2 cells were infected with HCoV-229E and HCoV-NL63, respectively, in the presence of the HTCC-62/63 or HTCC-63/77 mixture (1:1 w/w ratio, 50 μg/ml each), or control medium. Virus replication in cell culture supernatants was evaluated using quantitative RT-PCR on day 6 post-infection. Data are presented as Log Removal Value (LRV) compared to untreated samples. The assay was performed in triplicate, and average values with standard errors (error bars) are presented. * *P* < 0.05.

This study showed that HTCCs with different DSs function as potent inhibitors of all four LP-HCoVs. However, the HTCC antiviral activity against different pathogens varied depending on the percentage of amine groups substituted with ammonium groups in the polymer chain, i.e., the DS of the polymer. For example, HCoV-NL63 infection was effectively inhibited by HTCC-63 and HTCC-65, whereas HCoV-229E replication was significantly reduced by HTCC-62 and HTCC-77. To analyze whether these two pathogens could be inhibited using a single HTCC preparation, we tested the inhibitory effect of HTCC-62/63 and HTCC-63/77 mixtures on HCoV-NL63 and HCoV-229E infection. The results of *in vitro* study using LLC-Mk2 and MRC-5 cell lines demonstrated a significant decrease (an approximate 4 log reduction compared to the control sample) in HCoV-NL63 and HCoV-229E virus yields in the presence of HTCC-62/63 or HTCC-63/77 mixtures (1:1 w/w) (Fig 6).

## Discussion

Currently, no vaccines or antiviral drugs are available to prevent or treat HCoV—related diseases, including the life-threatening diseases resulting from SARS-CoV and MERS-CoV infection. Considering the severity of infections caused by the aforementioned pathogens and the prevalence of the remaining four HCoVs, the development of an effective antiviral treatment is of great importance. We showed previously that HTCC-63, a cationically modified chitosan and its hydrophobically-modified derivative are potent antiviral agents against HCoV-NL63. Both polymers also inhibited MHV infection *in vitro*, suggesting that they have an even broader antiviral activity spectrum.

Chitosan—a linear hetropolysaccharide derived from chitin was previously shown to have anti-inflammatory properties, and its use for drug delivery and tissue regeneration was proposed [20–22]. Further, chitosan has antibacterial activity against *Porphyromonas gingivalis*, *Aggregatibacter actinomycetemcomitans*, *Actinobacillus actinomycetemcomitans*, *Streptococcus mutans*, *Pseudomonas aeruginosa* and *Staphylococcus aureus* [23–27].

In the current study, we analyzed the mechanism of the HTCC antiviral activity. A thorough analysis revealed that the polymer does not affect virus attachment to target cells *via* HSPs, but effectively blocks subsequent interaction with the cellular receptor. Obtained results are consistent with our previous report, where we demonstrated a strong interaction between the HTCC-63 polymer and the recombinant ectodomain of the S protein of HCoV-NL63 [10].

Importantly, in the current manuscript, the previously described HTCC polymer substituted at DSs ranging from 57% to 77% successfully inhibited infection by all tested LP-HCoVs. The inhibitory activity of the polymers was evaluated *in vitro* using permissive cell lines (except for HCoV-HKU1) as well as *ex vivo* in primary HAE cultures. Unfortunately, due to the lack of an appropriate animal model for LP-HCoVs, *in vivo* analysis is not possible at present.

The results of these analyses demonstrated that HTCC-63 and HTCC-65 are potent inhibitors of both HCoV-NL63 and HCoV-OC43. On the other hand, polymers with DS of 62 and 77 proved very effective as inhibitors of HCoV-229E and HCoV-HKU1. Surprisingly, although HCoV-NL63 and HCoV-229E are close relatives, they were inhibited by polymers with different DS. Similarly, HTCC-63 hampered replication of three evolutionarily distinct viruses: HCoV-NL63, an Alphacoronavirus, and HCoV-OC43 and HCoV-HKU1, which belong to the Betacoronavirus genus. Data analysis shows that inhibition of each virus requires an HTCC with an optimal DS, suggesting that HTCCs need to be fine-tuned to interact with the viral fusion protein.

In conclusion, this study clearly shows that the polymeric compounds based on chitosan tested here efficiently inhibit infection by the HCoVs: HCoV-229E, HCoV-OC43, HCoV-NL63, and HCoV-HKU1. Interestingly, using HCoV-NL63 as the model system we determined that HTCC polymer blocks the interaction between the S protein and the cellular receptor, consequently hampering virus infection. The fact that polymers with different substitution levels are active against different CoVs suggest that these compounds interact with the S protein of different viruses, and may be optimized to be active also against other species. Thus, HTCCs hold great promise as anticoronaviral agents for use in the treatment of respiratory tract infections. Importantly, chitosan and chitosan-based polymers are not toxic *in vivo*, as shown by Yeh *et al* [28]. Further, Zeng *et al* demonstrated that HTCC is rapidly absorbed from gastrointestinal tract. The polymer was mainly distributed to lung, heart and kidney, whereas 24 hours later it was fully eliminated. These properties make HTCC a very promising compound for the use in clinic [29].

The potential of HTCCs is highlighted by the fact that the active substance of the first broad-range medicinal product approved for the treatment of respiratory infection is in fact a polymer (iota-carrageenan) [30–32]. It is worth noting, however, that this polymer is not effective against CoVs (unpublished data). The development of HTCC-based therapeutics may offer a solution.

## Supporting Information

S1 Fig Cytotoxicity of HTCC-63 in HAE cultures. Cell viability was assessed via XTT assay. Data on the y-axis represent the percentage values obtained for the untreated reference sample. Average values with standard errors are presented.
(TIF)
