# Supplementary figures and images for: HTCC: Broad Range Inhibitor of Coronavirus Entry
Source: PLoS One. 2016 Jun 1;11(6):e0156552. doi: 10.1371/journal.pone.0156552 (PMC4889042; doi:10.1371/journal.pone.0156552)

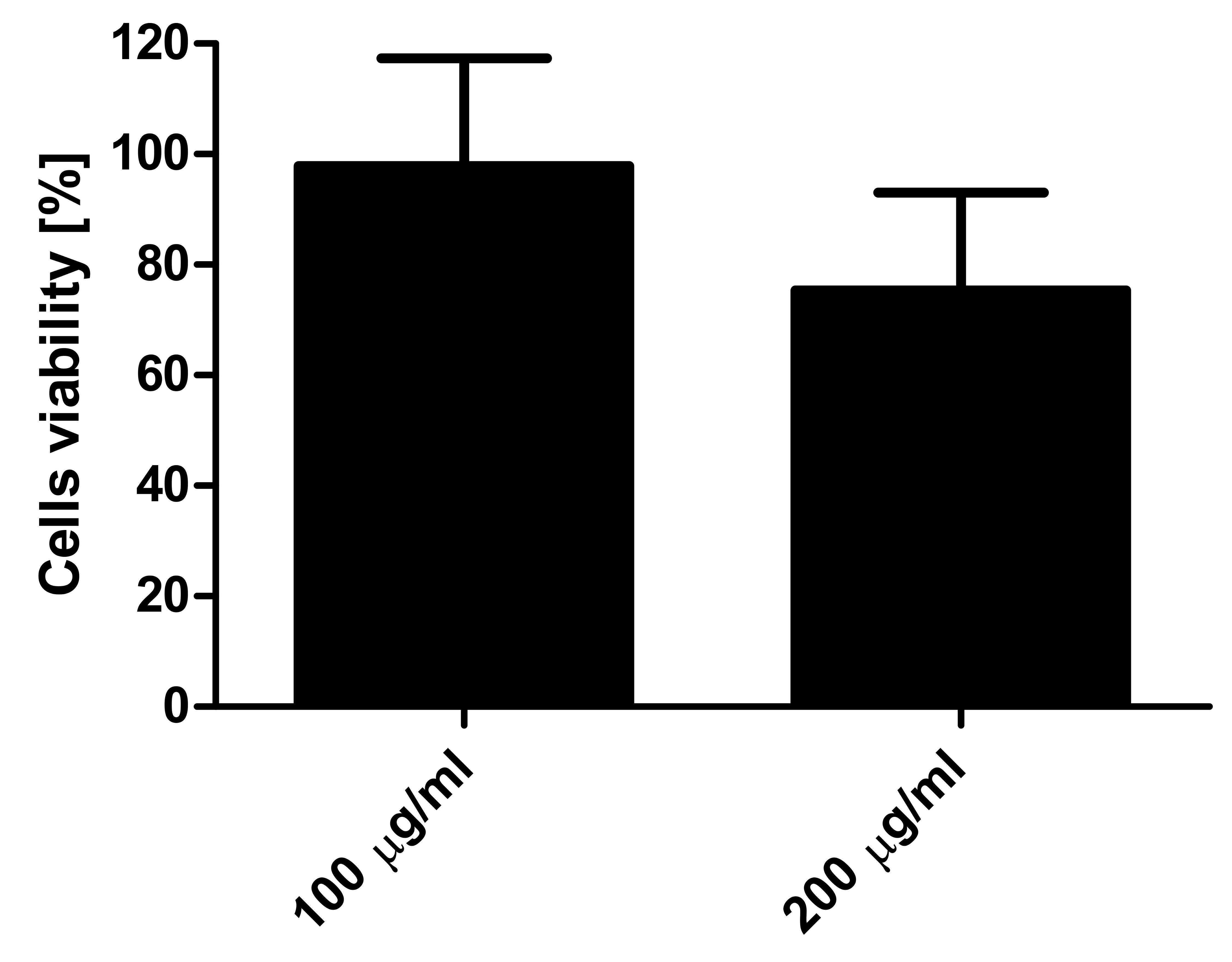

Supplement: S1 Fig — Cell viability was assessed via XTT assay. Data on the y-axis represent the percentage values obtained for the untreated reference sample. Average values with standard errors are presented. (TIF) [file pone.0156552.s001.tif]
